# Healthcare professionals’ perspectives on implementing opt-out smoking cessation in perioperative and cancer care in the Netherlands: A qualitative interview study

**DOI:** 10.18332/tpc/240141

**Published:** 2026-11-04

**Authors:** Iris Kramer, Annephine H. Cahn, Naomi A. van Westen-Lagerweij, Sophie J.A. Jooren, Esther A. Croes

**Affiliations:** 1 The Netherlands Expertise Centre for Tobacco Control, Trimbos Institute Utrecht The Netherlands

**Keywords:** smoking cessation, opt-out referral, implementation, preoperative care, cancer care

## Abstract

**Introduction:**

Smokers face increased risks of complications and mortality after surgery or cancer treatment. Smoking cessation care is usually offered in an opt-in manner, meaning that patients must actively choose to receive support. Consequently, support may primarily reach patients who are already highly motivated to quit. Combined with healthcare professionals’ barriers, such as limited time or training, this may reduce delivery of cessation care. An opt-out approach, in which healthcare professionals routinely refer all patients who smoke to smoking cessation support as part of standard care unless they decline, may increase uptake of cessation support. This study explored healthcare professionals’ perspectives on implementing opt-out smoking cessation care during the perioperative and cancer treatment phases.

**Methods:**

A qualitative study was conducted in the Netherlands using semi-structured interviews with healthcare professionals from different hospitals between March and April 2025. Eligible participants were healthcare professionals employed in a Dutch general, academic, or specialist oncology hospital and involved in perioperative or cancer care. The interviews were audio-recorded, transcribed verbatim, and analyzed using a thematic analysis approach combining deductive and inductive coding.

**Results:**

Thirteen healthcare professionals from ten Dutch hospitals were interviewed. Two overarching themes were identified: 1) perceived effects of the opt-out approach on patient participation and the doctor–patient relationship; and 2) facilitators and barriers to implementation. Participants anticipated increased uptake of smoking cessation support by lowering participation barriers. Facilitators included standardized referral pathways and routine integration. Potential challenges included limited time, staff constraints, patient motivation, and autonomy concerns.

**Conclusions:**

Healthcare professionals generally perceived the opt-out approach as an acceptable and potentially feasible strategy to improve access to smoking cessation support. Successful implementation is likely to require adequate organizational support and clear referral pathways. Further research is needed to identify optimal implementation strategies and evaluate impacts on referral rates, uptake of support, and patient outcomes.

## Introduction

Smoking remains a leading cause of preventable deaths worldwide^1^. Surgery or cancer treatment can serve as a ‘teachable moment’ in which patients are more motivated to quit smoking and more likely to remain abstinent post-treatment^2,3^. Numerous studies have shown that smokers face a higher risk of complications and mortality following surgery^4,5^, while preoperative smoking cessation significantly improves surgical outcomes^4,6^. Similarly, smoking cessation during cancer treatment (e.g. chemotherapy, immunotherapy, and radiotherapy) reduces side effects and enhances treatment effectiveness, resulting in higher survival rates^7,8^.

Healthcare professionals play a crucial role in initiating conversations about smoking cessation and referring patients to smoking cessation care^9,10^. In practice, smoking cessation care is typically offered in an opt-in manner, meaning that patients must actively consent to be referred to smoking cessation care. As a result, smoking cessation support may primarily reach patients who are already motivated to quit^11^. Besides, healthcare professionals experience barriers to discussing smoking cessation, such as lack of time or training^10^.

Implementing an opt-out approach may help overcome these barriers. With opt-out, patients are automatically referred to smoking cessation care when undergoing surgery or cancer treatment, unless they explicitly decline. Literature provides suggestive evidence that the implementation of an opt-out referral system leads to more applications for smoking cessation care and increases cessation attempts^11-15^, resulting in improved cessation outcomes^16^. For example, Himelfarb-Blyth et al.^12^ showed that an opt-out approach led to more healthcare professionals screening patients for tobacco use and recommending a cessation referral. In addition, patients were more likely to accept referrals in the opt-out approach (34.7%) compared to opt-in (11.5%). Richter et al.^11^ showed that the use of cessation medication increased with the opt-out approach (60% compared to 34%), and that more patients completed at least one counseling session (89% compared to 37%).

Despite promising outcomes in increasing patient participation in smoking cessation care, little is known about the extent to which an opt-out approach can be successfully implemented in practice. For instance, this approach has not yet been implemented in the Netherlands for smoking cessation. As healthcare professionals are the key actors in the delivery of the opt-out approach, it is important to first explore the perceptions of healthcare professionals regarding this approach. Therefore, the aim of this study was to explore the perspectives of healthcare professionals in the Netherlands regarding the implementation of opt-out smoking cessation care during the perioperative and cancer treatment phases. Understanding these perspectives represents an important first step in evaluating the feasibility and potential benefits of an opt-out approach. We explored key aspects, including the current integration of smoking cessation into perioperative and cancer care, and the barriers and facilitators of an opt-out system.

## Methods

### Study design and participants

A qualitative study was conducted between March and April 2025 among healthcare professionals working in Dutch general, academic, and specialist oncology hospitals to explore perspectives on the implementation of an opt-out smoking cessation approach. Semi-structured interviews and thematic analysis enabled an in-depth exploration of their experiences and views on the opt-out approach.

Participants were recruited using purposive sampling. Healthcare professionals were identified through the professional networks of the research team and collaborating healthcare professionals. Invitation emails were distributed directly and, in some cases, forwarded to colleagues who met the eligibility criteria. To obtain a broad range of perspectives, we aimed to include participants from different professions, hospital types, and clinical settings, including healthcare professionals who were not directly involved in smoking cessation research. Participants were required to be employed at a Dutch hospital (general, academic, or specialist oncology hospital), and only one physician per specialty was included from each hospital.

Those who expressed interest received an information letter along with scheduling options for the interview, which could be conducted either online or in person, based on their preference. Recruitment continued until thematic saturation was considered to have been reached. Following the final interviews, the research team determined that no substantially new themes or perspectives were emerging, suggesting that additional interviews were unlikely to generate novel insights relevant to the study aim.

### Procedures and measures

The interview protocol was developed by the study team, based on existing literature on smoking cessation care and the opt-out approach^9-11,14,17^ and supplemented by the team’s professional experience. The semi-structured interviews explored several themes, including the current smoking cessation care systems in the hospitals, perceived advantages and disadvantages of the opt-out approach, the impact on the doctor-patient relationship, and potential barriers and facilitators to implementing the opt-out approach.

Prior to discussing participants’ views on opt-out smoking cessation care, the interviewer provided a standardized explanation of the concept (Supplementary file Material 1). Participants were informed that opt-out smoking cessation care refers to an approach in which healthcare professionals routinely initiate referral to smoking cessation support for all patients who smoke as part of their perioperative or cancer care pathway, unless patients explicitly decline. Unlike the current opt-in approach, referral is presented as the standard pathway of care rather than requiring patients to actively request support. To facilitate understanding, the concept was illustrated using the Dutch organ and tissue donor registration system for donation after death, in which individuals are registered by default while retaining the option to opt out.

The interviews were conducted between March and April 2025 by a final-year medical student (AHC), who had previous experience conducting research and interviewing healthcare professionals. The interview guide was pilot-tested with a healthcare professional and reviewed by the research team before data collection, after which minor refinements were made. No additional formal training in qualitative interviewing was provided specifically for this study. Throughout data collection, interviews and emerging methodological issues were regularly discussed with experienced members of the research team, including researchers with expertise in qualitative research and smoking cessation care.

The interviewer had no prior relationship with the participants before recruitment, and participants were informed about her role in the study. No individuals other than the interviewer and participant were present during the interviews. All but one interview was conducted online via Microsoft Teams and lasted between 18 and 35 min. Interviews were recorded using an offline audio recorder and stored in a secure online environment. No field notes were taken during or after the interviews. Only the study team had access to the participants’ data. Names and other information that could potentially identify participants were removed, leaving only the professional title. Interview transcripts and preliminary findings were not returned to participants for comment or verification.

Throughout the analytical process, discussions were held within the research team to reflect on potential assumptions and preconceptions and their influence on data interpretation. The study was conducted in accordance with the principles of the Declaration of Helsinki and relevant institutional and national guidelines. The Ethics Committee of the Trimbos Institute determined that formal ethical review was not required for this study. Prior to each interview, participants received an information letter explaining the study and data management. Participants were given sufficient time to ask questions about the study before obtaining verbally informed consent at the start of each interview. The reporting of this study was informed by the Consolidated Criteria for Reporting Qualitative Research (COREQ).

### Coding and analysis

The audio recordings were transcribed verbatim using Amberscript. The transcripts were analyzed using a thematic analysis approach combining deductive and inductive coding. Deductive codes were derived from the topics addressed in the interview guide, while additional codes were developed inductively from the interview data during the analysis process. Codes were subsequently compared, refined, and grouped into broader patterns and overarching themes. The analysis was conducted using MAXQDA 24 (VERBI Software, Berlin, Germany). One researcher (AHC) coded all interviews. To enhance the trustworthiness of the findings, investigator triangulation was applied by involving a second researcher (SJAJ) in the review of the coding process after approximately half of the interviews had been coded and again after completion of all coding. Differences in interpretation were discussed until consensus was reached, and the coding framework was iteratively refined throughout the analysis. This resulted in a final coding framework comprising the main themes and their underlying categories (Supplementary file Figure S1). The main themes presented in the results section, together with illustrative quotations, were derived from this coding framework. The included citations were translated from Dutch into English.

## Results

Thirteen healthcare professionals from ten different hospitals were interviewed. Table 1 provides descriptive information about the participants, showing a broad variability in gender, hospital type, and medical profession. None of the participants worked in a hospital where an opt-out approach was already applied for smoking cessation care in the perioperative and cancer treatment phases. Five participants reported that their hospitals had a smoking cessation outpatient clinic, while others had to refer their patients to an external smoking cessation organization or general practitioner. All participants reported having experience discussing smoking with patients in routine clinical practice, but some participants were unaware of the various referral options. In addition, seven participants indicated that they did not consistently discuss smoking cessation due to time constraints and competing priorities.

**Table 1:** Characteristics of healthcare professionals participating in the qualitative interviews on the implementation of opt-out smoking cessation in perioperative and cancer care, the Netherlands, March–April 2025 (N=13)

Characteristics	n	%
**Gender**		
Male	7	53.8
Female	6	46.2
**Hospital**		
Academic	5	38.5
General	7	53.8
Oncology Center	1	7.7
**Treats patients during**		
Surgery	5	38.5
Chemotherapy	4	30.8
Radiotherapy	3	23.1
No physical treatment	1	7.7
**Profession**		
Radiotherapist	3	23.1
Internist	2	15.4
Surgeon	2	15.4
Anesthesiologist (resident)	2	15.4
Pulmonologist	1	7.7
Urologist	1	7.7
Nurse specialist oncology	1	7.7
Smoking cessation consultant	1	7.7

### Thematic analysis

We identified two overarching themes reflecting participants’ perspectives on the opt-out approach. The first theme highlights professionals’ views on how patients will respond to the opt-out approach, considering its impact on patient participation and its influence on the doctor–patient relationship. The second theme focuses on professionals’ own perspectives regarding implementation, including facilitators and barriers associated with its implementation.

### Theme 1: Healthcare professionals’ expectations of patients’ responses to the opt-out approach

Of the thirteen participants interviewed, twelve expressed support for the opt-out approach. Overall, participants viewed the approach as a valuable addition to current practice, with their support closely linked to expectations about how it could influence patient behavior. Specifically, participants emphasized that the opt-out approach could reinforce the importance of smoking cessation during these phases and increase patients’ awareness of their own responsibility throughout treatment:

*‘I think a lot still goes wrong for many patients, that they generally really have the feeling that they can just come and get something, and there’s little attention for the fact that patients themselves also have a big role to play in getting through it as well as possible.’* (Anesthesiology resident)

While most participants were supportive of the opt-out approach, variations in their understanding of the concept influenced how they anticipated patients would respond. In particular, three participants appeared to interpret the opt-out approach as limiting patients’ freedom of choice, assuming that patients would not be able to decline smoking cessation care. This interpretation does not fully align with the definition of an opt-out approach, in which patients retain the possibility to decline. Nevertheless, this perception led to concerns that patients might feel pressured or experience reduced autonomy, potentially affecting their engagement with the intervention.

#### Effect on inclusion of patients

Participants anticipated that standardizing the opt-out approach would result in higher uptake of smoking cessation support compared to the opt-in approach. Moreover, it would eliminate the need for patients to take proactive steps to access smoking cessation support:

*‘An opt-out approach, of course, requires some action [active refusal] from the patient. So it may be that patients are more likely to participate if things are arranged for them, so to speak, rather than having to take the initiative themselves.’* (Urologist)

Despite the generally positive attitudes, one participant expressed strong reservations. This participant worked in a hospital where smoking cessation care was already well integrated into routine clinical practice. In this context, the participant perceived the existing referral-based (opt-in) approach as effective and questioned the added value of implementing an opt-out strategy. Specifically, concerns were raised that an opt-out approach might lead to the referral of patients without intrinsic motivation to quit smoking, which could in turn increase the likelihood of premature dropout from smoking cessation programs:

*‘It turns out that a large proportion of the patients referred to the smoking cessation clinic never answer their phones again or respond in any way. So, if you tell someone, “You're going to have surgery, so I'm referring you to a smoking cessation clinic”, the clinic may spend a great deal of time calling and emailing these patients, only to hear nothing back.’* (Pulmonologist)

#### Effect on doctor–patient relationship

Three participants anticipated a beneficial effect of the opt-out approach on the doctor–patient relationship. They argued that the standardized nature of the opt-out approach reduces the perception that doctors are personally responsible for initiating the conversation, thereby minimizing blame. This could allow healthcare professionals to adopt a more supportive, less authoritative role:

*‘You're framing it in a more positive way. In this trajectory, we’re going to focus on smoking cessation. That’s simply part of it. And we’re going to help with that.’* (Internist 2)

In contrast, six participants suggested that the opt-out approach could potentially provoke a counterproductive response from unmotivated patients, with one participant hypothesizing that it might adversely impact the doctor–patient relationship.

Five participants emphasized that, given the substantial amount of information patients receive within a short timeframe, it is important that smoking cessation does not become overwhelming. They highlighted the need for clear and effective communication, both during clinical consultations and through information provided in advance. Participants stressed that patients should retain the option to decline smoking cessation care, and that maintaining a sense of autonomy is essential. Others similarly noted that the ability to opt out ensures that patient autonomy is preserved and that the doctor–patient relationship remains intact:

*‘I think it depends on how you frame it. If you say: for everyone starting chemotherapy who still smokes, we schedule an appointment to discuss smoking cessation, then I don’t think that puts any pressure on the doctor–patient relationship. Especially if someone still has the space to say, “Nice that you're offering this, but I’m not going to quit”. Then you're done, so to speak. People always have the freedom to say no, I think.’* (Internist 1)

### Theme 2: Healthcare professionals’ perspectives on implementing the opt-out approach

#### Facilitators for implementation

Participants indicated that implementing an opt-out approach during the perioperative and cancer treatment phases could support the integration of smoking cessation care into routine practice. Several participants noted that smoking cessation is not consistently discussed or followed up with referrals in the current opt-in system, and that an opt-out approach could help make these practices more routine:

*‘I think it [opt-out] would make things easier, because it would become a standard part of the procedure. Currently, I feel like I have to consciously remember to bring it up and make sure I don’t forget to ask.’* (Nurse specialist oncology)

Closely related to this, participants emphasized that standardizing the referral process across the hospital, and ideally at a national level, would further support embedding smoking cessation care into routine practice.

To improve time efficiency during the consultation and facilitate the discussion, participants noted that providing information to patients before the consultation would be beneficial. Furthermore, referrals to smoking cessation care should be straightforward and time-efficient. Ideally, the referral process would be fully integrated into the electronic health record, allowing appointments to be scheduled with minimal effort. Participants stressed that, particularly in the context of upcoming surgery or cancer treatment, a rapid and streamlined referral process is essential:

*‘What I would not prefer is that I need to go into different systems, with all kinds of things, where I have to manually retype the patient number, citizen service number, phone number, and so on. The ideal situation would be if I could just tick a box in the electronic health record and the process would continue automatically.’* (Radiotherapist 1)

Participants further highlighted that smoking cessation support should be easily accessible, for example through outpatient clinics, telephone consultations, or external providers. They emphasized the need for a clearly defined smoking cessation program for both healthcare professionals and patients, in which smoking cessation specialists take a proactive role in initiating contact:

*‘And I think that if it’s just a website where people have to register themselves, they might simply say, “Well, I’m not going to do that”. But if it’s a nurse who actively gets in touch with them … then it becomes much harder for people to just say, “I’m not doing that”.’* (Internist 2)

Participants expressed different views on whether smoking cessation care should be provided within the hospital or by external institutions. Advantages of in-hospital care include the ability to combine appointments with medical consultations and the motivational benefits of face-to-face interactions. Additionally, access to patients’ smoking status within the electronic health record was considered valuable for supporting ongoing encouragement. However, three participants noted that multiple hospital visits could be burdensome for patients, particularly those traveling long distances, and that external or online services may therefore be more appropriate in certain cases.

Finally, participants emphasized that providing healthcare professionals with additional knowledge and guidance would facilitate the implementation of the opt-out approach. This included knowledge about the effects of smoking on treatment outcomes, as well as practical guidance on how to communicate the opt-out approach effectively. Seven participants suggested that a concise, targeted form of training would be sufficient, rather than a comprehensive training program:

*‘So, I think mainly training focused on knowledge. So, how much of an impact does it have? How do you approach someone? Especially if they dig their heels in at first. ... Communication skills and knowledge, knowledge about how smoking affects your cancer treatment, the risk of recurrence of illness, and overall lifestyle.’* (Nurse specialist oncology)

#### Barriers for implementation

Participants identified several barriers that may hinder the implementation of the opt-out approach. A key concern was the limited time available during consultations. While participants agreed that responsibility for discussing smoking cessation should ideally lie with the treating specialist, time constraints often made this challenging, leading some to advocate for a greater role for nurse specialists:

*‘I sometimes must see up to eighty patients in a single day, and it [discussing smoking cessation] simply takes a lot of time. … So, who should do it? I think a nurse specialist would be perfectly suited for this. For example, after a doctor briefly informs the patient … saying, “You will soon undergo surgery, and we want you to stop smoking. I will refer you to the nurse specialist, who can provide further support”.’* (Anesthesiologist)

Participants reported that the priority given to smoking cessation discussions varied according to the clinical context. Smoking cessation was more likely to be prioritized when participants expected smoking to substantially affect treatment outcomes, recovery, or complication risk. Consequently, discussions were often more extensive in situations involving major surgery, high complication risks, or intensive cancer treatment trajectories.

Views on the impact of an opt-out approach on consultation time and communication were mixed. While some participants anticipated greater efficiency, others were concerned that a more streamlined referral process could reduce opportunities for meaningful patient communication and leave patients insufficiently informed about the referral process:

*‘Then it becomes just one sentence in a consultation of half an hour or three quarters of an hour with a lot of information for the patient. So, it may not really land.’* (Radiotherapist 1)

Some participants worried that a highly streamlined referral process could make communication more impersonal and reduce opportunities for questions, discussion, and patient engagement.

Participants also anticipated that the opt-out approach could increase the number of referrals to smoking cessation services, although they acknowledged that increased referrals would not necessarily translate into treatment uptake. Several participants reported existing waiting lists and capacity constraints within smoking cessation services, and some noted that local smoking cessation clinics had previously been discontinued because of funding constraints:

*‘The smoking cessation clinic here has such a long waiting list that by the time patients can be seen, 3 months may have passed.’* (Nurse specialist oncology)

These capacity limitations were perceived as a potential barrier to successful implementation, as increased referrals may not result in timely access to support if service capacity is insufficient.

## Discussion

In this study, we explored healthcare professionals’ perspectives on implementing an opt-out approach for smoking cessation care during the perioperative and cancer treatment phases. Overall, participants expressed support for the opt-out approach and expected it to facilitate the integration of smoking cessation into routine care. However, our findings also highlight that successful implementation depends not only on structural conditions, but importantly on healthcare professionals’ perceptions regarding patient motivation, communication, and feasibility.

### Interpretation of key findings

Most participants anticipated that the opt-out approach would increase patient participation by normalizing smoking cessation as a standard component of care. This aligns with previous research demonstrating that opt-out approaches increase referral uptake and smoking cessation attempts^11-13,15,18^. For example, Campbell et al.^18^ reported that the number of pregnant women who successfully quit smoking doubled after the introduction of the opt-out approach. These findings support the assumption that shifting from an opt-in to an opt-out approach can lower barriers to engagement and promote more consistent delivery of smoking cessation care.

Participants expressed different expectations regarding the impact of the opt-out approach on the doctor–patient relationship. While some anticipated resistance among unmotivated patients, others suggested that presenting smoking cessation as a routine aspect of care could reduce discomfort and support a more neutral, less directive interaction. These findings are consistent with the NICE review on opt-out approaches in prenatal care, which concluded that potential negative effects on the doctor–patient relationship are minimal when communication is clear and supportive^19^. Similarly, Campbell et al.^14^ found no adverse impact on the doctor–patient relationship following implementation of an opt-out approach. Together, this emphasizes the importance of communication strategies in shaping both patient acceptance and professionals’ experiences.

### Challenges

Despite generally positive attitudes towards the opt-out approach, some healthcare professionals expressed concerns that it could result in unnecessary referrals of patients who are not motivated to quit smoking. One participant suggested that the approach might reduce the quality of smoking cessation conversations by encouraging more automatic referrals. Notably, this participant worked in a hospital where smoking cessation care was already highly integrated into routine practice, with systematic assessment of smoking status and access to a dedicated outpatient smoking cessation clinic. Such a level of integration remains relatively uncommon in Dutch hospitals and may have influenced perceptions of the added value of an opt-out approach.

More broadly, concerns about referring unmotivated patients were linked to perceived inefficiencies and increased demands on healthcare professionals and smoking cessation services. Similar concerns have been reported previously^14,15,20,21^. These perceptions may be reinforced by findings showing that not all patients engage with cessation support following referral. For example, Nahass et al.^21^ found that only 49% of referred patients could be reached after opt-out inclusion, while Campbell et al.^14^ reported increased demands on staff time.

Although increased referrals do not always translate into immediate quit attempts, as shown by Bauld et al.^22^, opt-out approaches have been associated with greater engagement with smoking cessation services. In addition, evidence from other studies suggests that opt-out strategies may contribute to higher overall smoking cessation rates^21,23^. Restricting referrals to patients who appear motivated may therefore result in missed opportunities to engage smokers who would otherwise not seek support.

Furthermore, low initial motivation does not necessarily preclude successful participation, as motivational support is an integral part of Dutch smoking cessation care. Healthcare professionals may also underestimate patients’ readiness to quit during periods of illness or treatment. Surgical procedures are recognized as important teachable moments that can increase motivation to stop smoking^24^, while patients with cancer are generally more motivated to quit than smokers without a cancer diagnosis, especially when their cancer is related to smoking^25,26^. It remains unclear whether the effectiveness of the opt-out approach is specific to these settings or generalizable to all patients who smoke and receive hospital-based care.

Together, these findings suggest that a key barrier to implementation may be healthcare professionals’ perceptions regarding the value of referring patients with low motivation. Addressing these perceptions through education and training may therefore facilitate successful implementation.

### The need for a structured smoking cessation infrastructure

A key finding is that only a few of the hospitals where the participants were employed provided structured smoking cessation care. Participants highlighted the importance of having a well-established smoking cessation structure within the hospital, either through in-house support or clear referral options to external services. This underscores that effective implementation of the opt-out approach requires adequate smoking cessation infrastructure. In the Netherlands, a wide range of services is available through general practitioners and external providers, which hospitals can actively utilize through effective referral pathways.

To realize such a structure, hospitals need clear protocols outlining referral pathways to in-hospital clinics or external providers. Healthcare professionals should be aware of patients’ smoking status, ideally recorded in the electronic health record. As time constraints are a key barrier, efficient communication skills are essential, as highlighted by Shenton et al.^27^. The Very Brief Advice Plus method provides an example of effective brief communication, enabling proactive referral in three simple steps^28^. Evidence suggests that patients are up to 17 times more likely to engage in cessation programs when proactively referred^29^. However, participants’ limited awareness of referral options suggests that further training and improved communication are needed to support implementation.

### Patients’ perspectives

Although this study did not directly investigate patients’ perspectives, participants’ expectations align with existing literature. Sloan et al.^30^ and Ohde et al.^17^ showed considerable variation in patients’ responses to the opt-out approach. While some patients perceived the opt-out approach as a helpful incentive to quit smoking, others were not ready to quit. Ohde et al.^17^ found that patients often believed that smoking cessation would not significantly alter their treatment outcomes and that enduring the stress of trying to quit would not be worthwhile. Despite these differences in motivation, previous research suggests that patients generally expect healthcare professionals to address smoking during clinical encounters and regard this as a routine part of care^9^.

Taken together, these findings indicate the importance of clear, patient-centered communication about smoking cessation to support informed decision-making while respecting patient autonomy. As patient engagement is essential to the success of the opt-out approach, further research is needed to better understand patients’ perspectives.

### Strengths and limitations

To our knowledge, this is one of the first studies to explore healthcare professionals’ perspectives on implementing opt-out smoking cessation care across perioperative and cancer care settings in the Netherlands. Rather than focusing on patient outcomes, this study provides insight into anticipated implementation barriers, facilitators, and practical requirements for integrating opt-out smoking cessation care into routine clinical practice. These findings offer a foundation for future implementation and evaluation studies. Despite the small sample size, participants represented a diverse range of backgrounds and specializations. Data saturation was achieved, suggesting that key perspectives were effectively captured.

However, several limitations should be considered. Participants may have had a particular interest in smoking cessation care, potentially introducing selection bias. Although recruitment partly relied on professional networks, we purposively included healthcare professionals from different disciplines, hospital types, and clinical settings to obtain a broad range of perspectives. Nevertheless, information on non-participants was not systematically collected, and potential differences between participants and non-participants could therefore not be assessed. In addition, none of the participants had direct experience with implementing an opt-out smoking cessation approach in perioperative or cancer care. The findings therefore reflect healthcare professionals’ anticipated perceptions of implementation rather than experiences from actual practice, and some of the identified barriers and facilitators may differ from those encountered during real-world implementation. As we were not aware of any Dutch perioperative or cancer care settings with a formally implemented opt-out smoking cessation referral pathway, it was not possible to include healthcare professionals with direct implementation experience.

Furthermore, despite efforts to clearly explain the opt-out concept, some participants appeared to have an incomplete understanding of the approach during the interviews. Some participants incorrectly assumed that patients would not be able to refuse support under an opt-out system, whereas in practice patients always retain the right to decline. While clarifications were provided during the interviews, this misunderstanding may have influenced their responses and highlights the importance of clear definitions and communication when implementing such interventions in practice.

## Conclusions

This study shows that healthcare professionals generally support the opt-out approach and perceive it as a promising strategy for integrating smoking cessation care into routine clinical practice. Participants anticipated that its standardized nature could facilitate more consistent implementation and potentially improve patient participation in smoking cessation care. However, concerns remain regarding time constraints, resource availability, and the potential impact on patient motivation.

Successful implementation of the opt-out approach requires a well-structured and accessible smoking cessation care system. Hospitals should prioritize the development of clear protocols, efficient referral pathways, and sufficient capacity within cessation services. In addition, it is important to address healthcare professionals’ potentially limiting perceptions, such as concerns about referring patients with low motivation, as these may hinder implementation in practice. Future research should evaluate the implementation of the opt-out approach in practice, assess its effects on referral rates, treatment uptake, and patient outcomes, and examine in which patient populations the approach is most effective.
