# Supplementary material for: Healthcare professionals’ perspectives on implementing opt-out smoking cessation in perioperative and cancer care in the Netherlands: A qualitative interview study
Source: Tob Prev Cessat. 2026 Nov 4;12:10.18332/tpc/240141. doi: 10.18332/tpc/240141 (PMC13736799; doi:10.18332/tpc/240141)

TOBACCO PREVENTION AND CESSATION

Supplementary file

© 2026 Kramer I. et al.

DOI:
10.18332/tpc/240141

The content has been provided by the author(s) and has not been reviewed, verified, or endorsed by European Publishing. It may not have undergone peer review. The views, opinions, and recommendations expressed are solely those of the author(s) and do not necessarily reflect the position of European Publishing. European Publishing accepts no responsibility or liability for any consequences arising from the use of, or reliance on, this content.

SUPPLEMENTARY FILES

Supplementary File 1. Semi-structured interview guide

Before we start, we would like to explain what we mean by opt-out smoking cessation care. In this study, opt-out smoking cessation care refers to an approach in which healthcare professionals routinely refer patients who smoke to smoking cessation support as part of their perioperative or cancer care pathway, unless the patient explicitly declines. In contrast to the current opt-in approach, referral is presented as the standard pathway of care rather than something that patients must actively request. Patients remain free to decline support at any time. A comparable example is the Dutch donor registration system, in which individuals are included by default but may choose to opt out.

With this definition in mind, we would now like to ask about your views on implementing such an approach in your clinical practice.

Current healthcare system

1. Do you discuss smoking cessation with patients during the period surrounding surgery or cancer treatment? If so, at what point do you discuss it and what do you typically discuss?
2. **Physician / Nurse / Nurse specialist:**
    - Is there a service within your hospital to which patients can be referred for smoking cessation support?
    - If so, how does the referral process currently work?

   Smoking cessation consultant:
    - How often are patients referred to you for smoking cessation support during the perioperative period or cancer treatment trajectory?

Characteristics of an opt-out system

1. What do you see as the potential benefits or positive effects of implementing an opt-out smoking cessation approach?
2. What do you see as the potential disadvantages or negative effects?
3. How would you ideally organise an opt-out smoking cessation system within your healthcare setting?
    - At what point in the care pathway should it be discussed?
    - Who should discuss it?
    - Who should provide the smoking cessation support?

Communication with patients

1. Would an opt-out approach make conversations about smoking cessation easier or more difficult for you? Why?
2. How do you expect patients would react to an opt-out approach?

3. **Physicians / Nurses / Nurse specialists (excluding smoking cessation consultants)**: How do you think an opt-out approach might affect the healthcare professional-patient relationship?

Implementation process

1. What would be required to implement your ideal opt-out smoking cessation system?
    - What would be needed from a logistical perspective (e.g., a smoking cessation clinic, smoking cessation consultant, electronic referral system)?
    - Would healthcare professionals require additional training or education?
2. Is there anything else regarding opt-out smoking cessation care that we have not discussed but that you consider important?

Supplementary figure S1. Overview of the coding framework, categories, subcategories, and themes

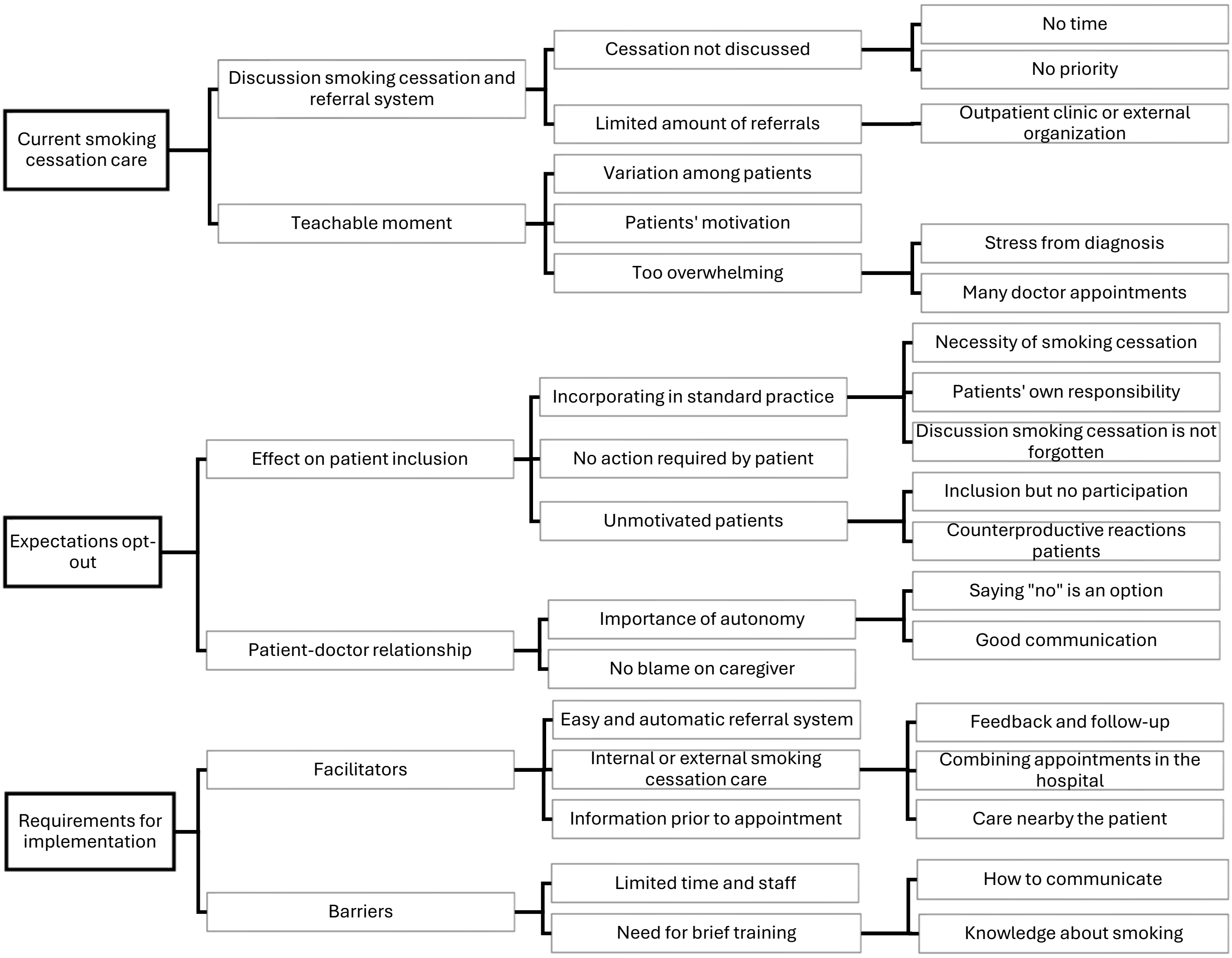

Supplement: Supplementary file 1 [file TPC-12-69-s001.pdf]
